# Reward Dependent Invigoration Relates to Theta Oscillations and Is Predicted by Dopaminergic Midbrain Integrity in Healthy Elderly

**DOI:** 10.3389/fnagi.2017.00001

**Published:** 2017-01-24

**Authors:** Tineke K. Steiger, Nico Bunzeck

**Affiliations:** ^1^Institute for Psychology, University of LuebeckLuebeck, Germany; ^2^Department of Systems Neuroscience, University Medical Center Hamburg-EppendorfHamburg, Germany

**Keywords:** dopamine, aging, reward, theta oscillation, substantia nigra

## Abstract

Motivation can have invigorating effects on behavior via dopaminergic neuromodulation. While this relationship has mainly been established in theoretical models and studies in younger subjects, the impact of structural declines of the dopaminergic system during healthy aging remains unclear. To investigate this issue, we used electroencephalography (EEG) in healthy young and elderly humans in a reward-learning paradigm. Specifically, scene images were initially encoded by combining them with cues predicting monetary reward (high vs. low reward). Subsequently, recognition memory for the scenes was tested. As a main finding, we can show that response times (RTs) during encoding were faster for high reward predicting images in the young but not elderly participants. This pattern was resembled in power changes in the theta-band (4–7 Hz). Importantly, analyses of structural MRI data revealed that individual reward-related differences in the elderlies’ response time could be predicted by the structural integrity of the dopaminergic substantia nigra (SN; as measured by magnetization transfer (MT)). These findings suggest a close relationship between reward-based invigoration, theta oscillations and age-dependent changes of the dopaminergic system.

## Introduction

The ability to anticipate and translate reward into appropriate actions is crucial for goal-directed behavior. In humans, reward motivation can accelerate response times (RTs; Knutson et al., 2001a), enhance physical effort (Pessiglione et al., 2007) and improve subsequent memory (Adcock et al., 2006). A prime candidate for such invigoration of behavior by reward is the dopaminergic system as suggested by computational models and empirical studies in animals and human subjects (Niv, 2007; Guitart-Masip et al., 2011; Dayan, 2012; Beierholm et al., 2013). Importantly, the impact of structural decline of the dopaminergic system during healthy aging remains unclear.

At the neuronal level, dopamine neurons of the substantia nigra (SN) and ventral tegmental area (VTA) fire in response to reward and thereby release dopamine into the nucleus accumbens (NAcc; Schott et al., 2008; Haber and Knutson, 2010). During Pavlovian conditioning, a cue predicts a subsequent reward and the neuronal response, that is initially observed to the delivery of the reward, shifts to the reward-predicting cue, suggesting a role of the dopaminergic system in reward anticipation (Fiorillo et al., 2003). Finally, the SN/VTA and the NAcc are sensitive to reward magnitude, with higher rewards leading to more neural activation and more efficient behavior, such as faster RTs, in contrast to lower rewards (Knutson et al., 2001b; Tobler et al., 2005).

Physiologically, the SN/VTA is connected with the medial temporal lobe (MTL) via parts of the basal ganglia (including the NAcc) and hippocampal novelty signals elicit dopamine release directly back to the MTL, promoting long-term potentiation (LTP; Lisman and Grace, 2005; Haber and Knutson, 2010; Lisman et al., 2011). In support of this hippocampus-SN/VTA model, human studies could show that reward anticipation enhances memory via SN/VTA and NAcc activity (Wittmann et al., 2005; Adcock et al., 2006; Bunzeck et al., 2012).

During healthy aging, the dopaminergic system declines (Fearnley and Lees, 1991; Bäckman et al., 2006; Bunzeck et al., 2007), which may lead to changes in novelty processing and memory performance (Bunzeck et al., 2007; Düzel et al., 2008). Importantly, it may also account for the absence of reward anticipation in the elderly as found in some (Schott et al., 2007) but not all studies (Samanez-Larkin et al., 2007). In fact, using fMRI Schott et al. (2007) could show that healthy elderly exhibit the opposite pattern to young controls with absent mesolimbic reward prediction responses, but instead mesolimbic activation to the reward feedback. Although this suggests a direct relationship between structural integrity and reward anticipation in the elderly, empirical evidence remains unrevealed.

Using electrophysiology, studies in rodents and humans have linked reward anticipation to activity in the theta-band (4–7 Hz; Paz et al., 2008; van Wingerden et al., 2010; Bunzeck et al., 2011; Gruber et al., 2013). In general terms, theta oscillations may play a major role in various cognitive domains by synchronizing the neuronal activity across different brain areas, probably reflecting the coordination of processing relevant information (Buzsáki and Draguhn, 2004; Düzel et al., 2010). More specifically, during decision making, theta may serve as a mechanism to coordinate the retrieval of choice relevant information (e.g., stimulus-reward associations, or stimulus-stimulus associations) and to assess sensory input to initiate goal-directed behavior (Womelsdorf et al., 2010; Silvetti et al., 2014). In memory paradigms, power in the theta band prior to the onset of a to-be-learned stimulus is enhanced for stimuli that are remembered in later memory tests (Addante et al., 2011). Importantly, if theta power is increased by reward anticipation prior to a stimulus, subsequent memory performance improves (Gruber et al., 2013). While this mechanism is well established in young adults, the influence of aging on theta-based reward anticipation and its effect on long-term memory formation is largely unknown.

The aims of this study were, first, to investigate the effect of aging on reward related behavior and associated theta power, and, second, to further explore the influence of the elderlies’ inter-individual structural variance of the dopaminergic mesolimbic system on reward anticipation. We hypothesized, that anticipating high compared to low monetary reward would result in: (a) faster RTs and increased theta power during encoding and (b) improved subsequent memory performance in young participants. In the elderly, (c) we assumed no or less theta power and invigoration during reward anticipation due to structural declines of the mesolimbic dopaminergic system. Finally, (d) we expected theta power to be modulated in the elderly, but not in the young participants, when receiving the reward feedback.

## Materials and Methods

### Participants

A group of 22 young (11 males, mean age = 25.5 years, SD = 3.1 age-range 20–32) and 32 healthy elderly participants were tested. One elderly subject had to be excluded due to brain anomalies; the remaining 31 participants consisted of 14 males (mean age = 67.3 years, SD = 6.2 age-range 56–78). All (young and elderly) were right-handed, had normal or corrected-to-normal vision (including color-vision) and none of the participants reported any history of neurologic or psychiatric disorders, or current medical problems (excluding blood pressure).

The elderly successfully completed the Geriatric Depression Scale (GDS; mean GDS = 1.4, SD = 1.9, GDS ≤ 5 for all participants; GDS ranges from 0 to 15, scores higher than 10 indicate depression; Sheikh et al., 1991) and the neuropsychological battery of the “Consortium to Establish a Registry for Alzheimer Disease” (CERAD; Welsh et al., 1991) including the Mini Mental State Examination (MMSE; Folstein et al., 1975; mean MMSE = 29.5, SD = 0.77, MMSE ≥ 27 for all participants; MMSE ranges from 0 to 30, scores smaller than 25 indicate pathologies) to access mental well-being and cognitive integrity. Data from this group of elderly participants have already been published (Steiger et al., 2016). This study was carried out in accordance with the recommendations of the local ethics committee (Medical Association Hamburg). All subjects gave written informed consent in accordance with the Declaration of Helsinki. The protocol was approved by the local ethics committee (Medical Association Hamburg).

### Experimental Design

**Figure 1:**
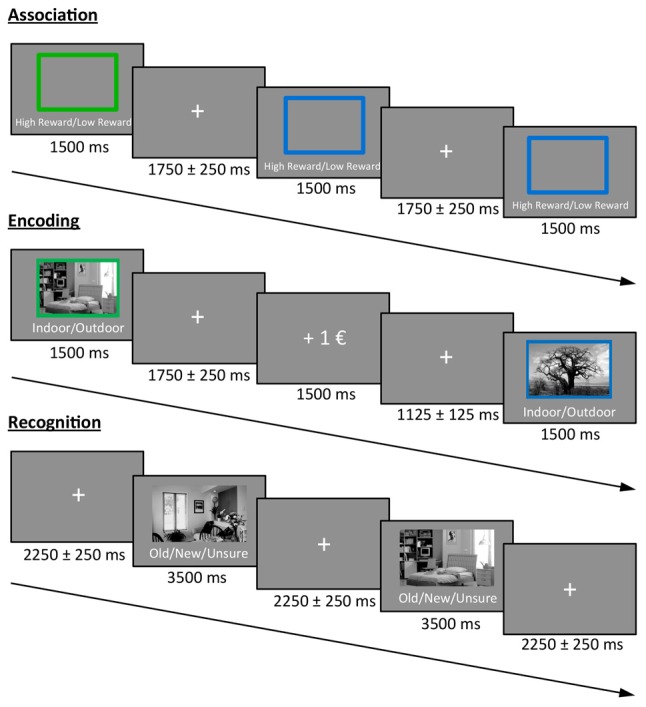
**Task design.** Participants first learned the color-reward association. During the encoding phase, participants had to indicate whether a presented stimulus was an indoor or outdoor image. The color of the frame predicted either a high or low reward for correct classification. During the recognition phase, images from the encoding phase were randomly intermixed with new images and participants had to indicate the old/new status (or to press a third button when unsure), without receiving any reward.

The experimental task was divided into three phases: association, encoding and recognition (Figure 1). In the first phase (association) participants were informed about the color-reward association and subsequently had to press two different buttons (indicating high vs. low reward) in response to a blue or green frame (10 frames; in this phase, no reward feedback was given). The color-reward association was counterbalanced across participants, and the association phase was followed by two short training blocks that were identical to the subsequent encoding and recognition phase. Therefore, encoding during the actual experiment was not incidental.

During the encoding phase, 30 indoor and 30 outdoor scene images (gray scaled) were presented for 1.5 s each in random order, with the prompt “Indoor/Outdoor” below the image. Accordingly, participants indicated the indoor/outdoor status of the stimulus by button press using their index and middle finger of the right hand. Importantly, during picture presentation, either a green or a blue frame surrounded the image and predicted a high or low reward for a correct indoor/outdoor classification. For each participant, the color reward magnitude association was identical to the association phase, and the images were randomly assigned to a frame. The direct feedback (“+1€” or “+0.1€” for correct classification, “X” for incorrect classification) was presented for 1.5 s after a jittered inter-stimulus interval (ISI) of 1.75 ± 0.25 s in which a fixation cross was presented at the center of the screen. The contingency between cue and reward outcome was 100% and participants were informed before the experiment that they will be paid part of their actual earnings at the end. During a 10 min break after encoding, participants had to answer geography questions in order to avoid memory retrieval from working memory in the subsequent recognition test.

During the recognition phase, the previously encoded 60 images were randomly presented and intermixed with 30 new images together with the prompt “Old/New/Unsure” below each image. In order to prevent guessing, participants were instructed to only press “old” or “new” if they were confident. The decision had to be indicated via button press (index, middle and ring finger of the right hand) during the presentation of the image (3.5 s), which was followed by a central fixation cross in a jittered ISI of 2.25 ± 0.25 s before the next image appeared.

All pictures were presented at the center of the screen with a size of 7.6° × 4.5° (visual angle). The surrounding frame had an additional size of 0.4°.

The encoding and recognition phase were repeated for a second time after a short break with new images, resulting in a total of 120 encoded images (60 high and 60 low rewarded) that were intermixed with 60 new images during retrieval. Participants were paid 12% of their total earnings in addition to their hourly reimbursement rate at the end of the experiment.

Importantly, during all tasks, subjects were instructed to respond as quickly and accurately as possible.

### Analysis of Behavioral Data

For the encoding phase, the median RTs of each participant for the indoor/outdoor classification of high and low reward images were calculated using MATLAB (The Math-Works Inc., version 2014b). The median was chosen because it is a measure that is less susceptible to outlying responses. The hit rate (percentage of correct indoor/outdoor classification) was taken as a measure of accuracy.

For the recognition phase, we analyzed median RTs for correctly remembered images and corrected hit rates (CHR) separately for images that were either high or low reward predicting during encoding. CHR were calculated by subtracting the false alarm rate (i.e., new images that were classified as old) from the hit rate (correct old classification) for each participant. Items classified as “unsure” were not included in the data analysis. All data were analyzed using IBM SPSS Statistics (version 21).

### EEG-Recording and Preprocessing

During encoding and recognition, electroencephalographic (EEG) activity was acquired with a 60-channel active electrode system positioned according to the 10-20 system using acticap (Brain Products GmbH, Munich, Germany) and Brain Vision Recorder (version 1.03.0003). FCz was used as a reference and the right mastoid as a ground electrode. In accordance with the manual and previous studies using active electrodes (Eckart et al., 2014, 2016) impedances were kept below 20 kΩ. To control for horizontal and vertical eye movements, two pairs of additional electrodes were used. Data were recorded with a sampling rate of 500 Hz and a high-pass (0.1 Hz) and low-pass (1000 Hz) filter.

Preprocessing of the EEG data was conducted using EEGLAB (version 13_4_4b; Delorme and Makeig, 2004) and customized MATLAB tools. First, the continuous data were high-pass (1 Hz) and low-pass (60 Hz) filtered; subsequently, all trials of the encoding phase were epoched from 600 ms before to 1400 ms after onset of the image- (or feedback-) presentation and downsampled to 250 Hz. This resulted in epochs for the following four conditions: high reward image, low reward image, high reward feedback and low reward feedback. Note that four of the young participants did not have epochs for the feedback-stimuli due to technical issues.

Subsequently, all trials were visually inspected to identify major atypical artifacts (mostly movements or muscle artifacts) and bad channels. Moreover, artifacts due to blinks or eye movements were removed using independent component analysis (ICA; Delorme and Makeig, 2004). Bad channels had to be interpolated in five participants (only one single channel for each subject). After this procedure, all epochs were visually inspected again and rejected when still containing artifacts. Finally, epochs were re-referenced to the average reference. After preprocessing, an average number of 54 trials per participant and condition remained (55 for high reward images, 55 for low reward images, 54 for high reward feedback, 53 for low reward feedback).

### EEG-Analyses

The analysis of the EEG data was conducted using Fieldtrip (version 2015-11-12; Oostenveld et al., 2010) with customized MATLAB scripts. Time-frequency decompositions were conducted from 4 Hz to 30 Hz using convolution on the single-trial time series with complex Morlet wavelets (four cycles) in steps of 0.5 Hz in the frequency- and 8 ms in the time-domain. Power was averaged across trials for each condition of interest. Subsequently, a baseline correction was applied using the condition-specific relative baseline (100–90 ms prestimulus). Note that this relatively short baseline comprises data points from a 570–1000 ms time window for the frequencies of interest (4–7 Hz) due to the temporal smoothing introduced by the 4-cycle wavelet transformation.

For analyzing power differences between conditions in the theta-band (4–7 Hz), non-parametric cluster-based permutation tests (Maris and Oostenveld, 2007) were conducted in a time window from 0 ms to 900 ms after stimulus onset to avoid edge effects. *T* tests were conducted for all contrasts on each individual sample and adjacent significant samples (*p* < 0.05) were clustered with the restriction of considering only effects significant on three or more neighboring channels. A Monte Carlo estimate of the permutation *p*-value was calculated to control for multiple comparisons. Condition labels were randomly permuted (*n* = 1000) and test statistics were computed again for every of these random partitions. The proportion of randomly drawn partitions resulting in larger test statistics than in the real data gave the *p*-value. Clusters with *p* < 0.05 were considered significant.

### Image Acquisition and Processing

We took advantage of structural MRI data for all elderly (but not young) participants acquired in a separately conducted study using a 3T MR system (Siemens Trio) with a standard 32-channel head coil as described in Steiger et al. (2016). Briefly, whole-brain multiparameter mapping (MPM; Draganski et al., 2011) was conducted on the basis of multi-echo 3D FLASH (fast low angle shot) images at 1 mm isotropic resolution with predominantly proton density (PD), magnetization transfer (MT) or *T*_1_ weighting. The total scanning time of MPM protocol was approximately 20 min.

The Statistical Parametric Mapping (SPM8) framework (Wellcome Trust Center for Neuroimaging, London) and customized MATLAB tools were used for data processing. The semi-quantitative parameter map of MT represents the percentage loss of magnetization induced by the MT saturation pulse and was calculated as described in Helms et al. (2008).

The MT maps were segmented into gray matter (GM), white matter (WM) and cerebrospinal fluid (CSF) for voxel-based morphometry (VBM) within the unified segmentation approach (Ashburner and Friston, 2000, 2005). MT maps were used for segmentation to help separating the effects of iron concentration from atrophy (Helms et al., 2009; Lorio et al., 2014). The GM images were non-linear transformed to standard Montreal Neurological Institute (MNI) space using the diffeomorphic registration algorithm (DARTEL) implemented in SPM8 (Ashburner, 2007), scaled by the Jacobian determinants of the deformation field and smoothed with an isotropic Gaussian Kernel of 6 mm full width at half maximum (FWHM). Due to their* a priori* rather low specificity, WM volume maps were excluded.

A voxel-based quantification (VBQ) analysis was used (Draganski et al., 2011) on the MT images to preserve quantitative parameter values, reducing effects of residual misregistration and partial volume effects and enhance tissue-specificity, allowing us to conduct analyses in WM and GM separately. The MT images were normalized into MNI space using the participant-specific deformation fields from the DARTEL procedure without modulating by the Jacobian determinant. Instead, a combined tissue-specific weighting/smoothing procedure (3 mm FWHM isotropic Gaussian smoothing kernel) was used (Draganski et al., 2011).

### Relationship Between Brain Structure and Behavior

Whole-brain linear regression models as implemented in SPM8 were used to investigate the relationship between the elderlies’ GM or MT maps, respectively, and their individual reward benefit on RT during encoding (i.e., RT low reward divided by RT high reward for each participant). Clusters with more than 25 voxel and a *p* < 0.05 after family-wise error correction at cluster-level were regarded significant.

**Figure 2:**
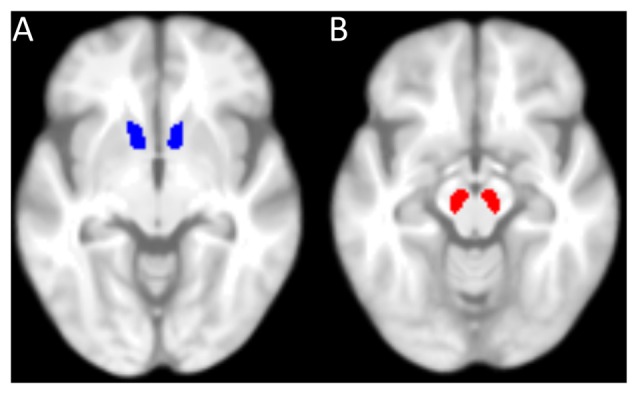
**Masks used for the region of interest analysis. (A)** 50%-probability mask of the nucleus accumbens (NAcc) from the Harvard-Oxford-Atlas (implemented in FMRIB Software Library, FSL). **(B)** Mask of the substantia nigra (SN)/ventral tegmental area (VTA), manually drawn. Both masks are superimposed on the mean T1-weighted image of all elderly participants.

Furthermore, on the basis of our* a priori* hypotheses (see “Introduction” Section), the SN/VTA and the NAcc were defined as regions of interest (ROIs). The mask for the NAcc (Figure 2A) was taken from the Harvard-Oxford-Atlas (50% probability mask), implemented in the FMRIB Software Library (FSL; Jenkinson et al., 2012). Since there was no SN/VTA-mask available in the Harvard-Oxford-Atlas, it was manually drawn in MRIcron (Rorden and Brett, 2000) on the basis of the mean MT-weighted (MTw)-images of the participants (Figure 2B). Mean GM and MT values were extracted from each participant’s ROI. Values from left and right hemisphere were averaged, since we had no hypothesis about laterality. Using IBM SPSS Statistics, the resulting values were entered into partial correlation analyses with reward benefit on RT during encoding, controlled for age. *P*-values were corrected for multiple comparisons (four comparisons: GM and MT in both ROIs) resulting in a significance threshold of *p* = 0.05/4 = 0.0125.

### Relationship Between Brain Structure and EEG Oscillations

EEG power differences between high and low reward during encoding were calculated for each participant using the statistical mask derived from the non-parametric permutation test (see “Result: Effect of Reward Anticipation on EEG Spectral Power” Section). Since there was no effect across all elderlies, the significant cluster from the younger subjects (see Figure 4A) served as mask to extract the mean power for the elderly. As described above, the mean power differences were used as regressors on the elderlies’ GM and MT maps, respectively, in a whole brain regression analyses and also in the subsequent ROI analyses. Finally, the derived power values were also used for correlations with individual reward related RT differences during encoding.

## Results

### Behavioral Data

**Figure 3:**
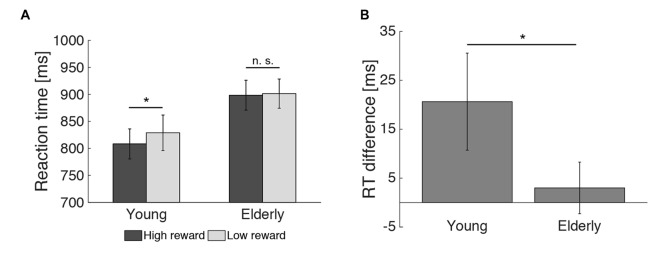
**Effect of reward on reaction times during encoding. (A)** The reward related difference in reaction times (RTs) during encoding was significant in the young, but not the elderly participants. **(B)** The RT difference for high vs. low reward predicting images differed between both age groups (*p < 0.05; n. s. = not significant: *p* > 0.05).

2 × 2 analysis of variance (ANOVAs) were calculated to investigate the effects of reward (high vs. low) and age group (young vs. elderly) on the behavioral data. In the encoding phase, both young and elderly participants performed with high accuracy in the indoor/outdoor classification (mean hit rate = 97.77%, see Table 1), with no main effect of reward (*F*_(1,51)_ = 0.091, *p* = 0.76) or age group (*F*_(1,51)_ = 0.008, *p* = 0.928) and no interaction of both factors (*F*_(1,51)_ = 0.091, *p* = 0.76). For RT, there was a significant main effect of reward (*F*_(1,51)_ = 5.130, *p* = 0.028), a marginally significant main effect of age group (*F*_(1,51)_ = 3.914, *p* = 0.053) and a trend for an interaction between reward and age group (*F*_(1,51)_ = 2.860, *p* = 0.097). To further examine this trend, two-tailed *t* tests comparing RTs for high vs. low reward were performed within the two groups separately. It revealed a significant effect only in the young (*t*_(21)_ = −2.08; *p* = 0.050) but not in the elderly (*t*_(30)_ = −0.57; *p* = 0.575, Figure 3A). Finally, a one-tailed *t* test was conducted between both age groups on the difference between RT for high vs. low reward. It revealed a significant difference between both groups (*t*_(51)_ = −1.69; *p* = 0.048, Figure 3B), which is in line with our* a priori* hypotheses of an effect of reward anticipation only in the young but not elderly subjects.

**Table 1:** **Behavioral data of the young and elderly participants for the encoding and recognition phase (mean values with standard errors, SE)**.

	Young		Elderly	
	High reward	Low reward	High reward	Low reward
*Encoding task*
Reaction time (ms) SE	808.30 (27.89)	828.93 (32.88)	898.43 (27.63)	901.43 (27.13)
Hit rate (%) SE	97.80 (0.52)	97.80 (0.57)	97.63 (0.56)	97.85 (0.46)

*Recognition task*
Reaction time (ms) SE	1290.82 (41.85)	1290.82 (43.14)	1309.64 (37.35)	1302.76 (44.14)
Corrected hit rate (%) SE	40.30 (3.21)	40.61 (3.19)	34.89 (3.36)	34.14 (3.46)

For the recognition memory task, there was no main effect of reward or age group on CHR and RTs, and no significant interaction between both factors (*p* > 0.05, see Table 1).

### Effect of Reward Anticipation on EEG Spectral Power

**Figure 4:**
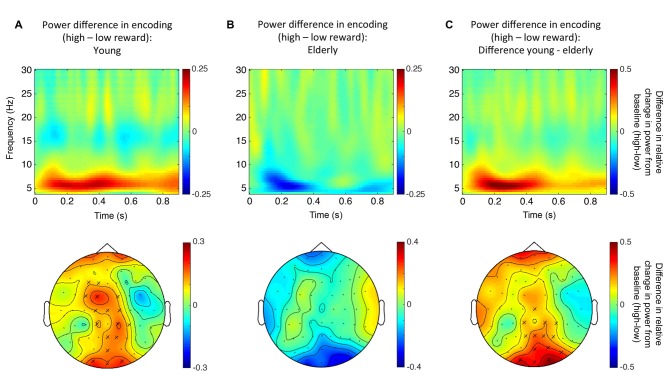
**Reward related theta power modulation. (A)** Top row: time-frequency plot for high vs. low reward predicting images for young participants (averaged over all electrodes that form the significant cluster). Bottom row: topographical distribution averaged over the theta-band in the significant time window (56–552 ms after stimulus onset). **(B)** Top row: time-frequency plot for high vs. low reward predicting images for the elderly (not significant; averaged over all electrodes that were part of the significant cluster in the young participants). Bottom row: topographical distribution averaged over the theta-band in the same time window as in panel **(A)**. **(C)** Top row: time-frequency plot of the difference in reward anticipation between young and elderly participants (see text; averaged over all electrodes that form the significant cluster). Bottom row: topographical distribution averaged over the theta-band in the significant time window (40–624 ms after stimulus onset). “X” indicates electrodes of the significant cluster.

A first Monte Carlo cluster-based permutation test revealed a significant difference (*p* = 0.038) between spectral powers of high vs. low reward predicting images in the theta-band (4–7 Hz) of the young participants. We found that theta power was increased in the high compared to the low reward condition. The effect lasted from 56 ms until 552 ms after stimulus onset and the cluster was widely distributed across occipito-parietal and fronto-central electrodes (17 electrodes in total; Figure 4A). In contrast, for the elderly subjects, neither a positive nor a negative effect was found in the theta-band (Figure 4B). To further quantify this potential age effect, the spectral difference was calculated for every participant by subtracting the power for low reward from high reward predicting images. Importantly, a subsequent Monte Carlo cluster-based permutation test revealed a significant difference (*p* = 0.026) between both groups for reward anticipation in the theta-band in the time range from 40 ms to 624 ms after stimulus onset at central and occipital-parietal electrodes (14 electrodes in total; Figure 4C). This pattern mirrors the RT effect (Figure 3).

For reward feedback, no statistically significant difference between high and low reward was observed, neither in the young nor in the elderly participants. Similarly, there were no significant correlations between reward related behavioral differences in RT and changes in theta power.

### Relationship Between Brain Structure and Reward Anticipation in the Elderly

**Figure 5:**
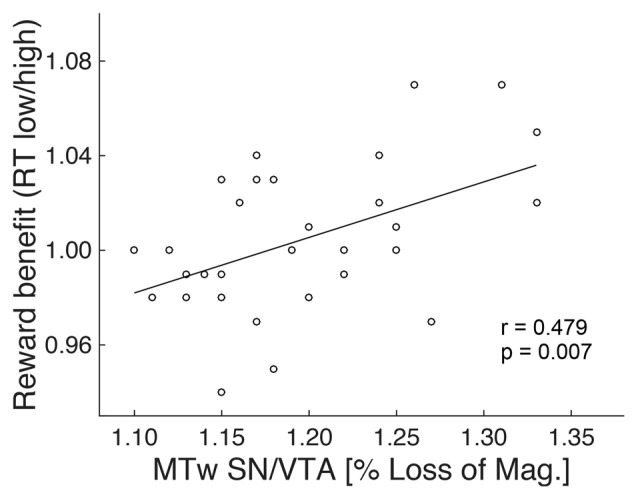
**Structure-behavior relationship.** The degree of RT benefit from reward during encoding was positively correlated with the magnetization transfer of the SN/VTA white matter (MTw; indicative of myelin) in the elderly.

To further investigate the absence of a reward anticipation effect in the elderly, MRI data of this group were analyzed (see “Material and Methods: Relationship Between Brain Structure and Behavior” Section). Specifically, the ratio between RTs of the two reward magnitude conditions during encoding (RT low reward divided by RT high reward) was taken as a measure of RT benefit from reward. It has been used as regressor in a whole brain analyses and was subsequently correlated with the structural markers (GM and MT) of the NAcc and SN/VTA, controlling for age (partial correlation). There was no significant correlation between RT reward benefit and MT or GM on a whole brain level, or with values extracted from the NAcc ROI. However, there was a significant positive correlation (*r* = 0.479; *p* = 0.007) between RT reward benefit and MT (but not GM) values of the SN/VTA WM (Figure 5).

The analyses of correlations between reward-related differences in the elderlies’ EEG signal and structural markers revealed no significant effect (*p* > 0.05).

## Discussion

We examined the neural mechanisms of reward processing and its relationship to healthy aging. In line with our hypotheses, only young participants benefited from reward anticipation during encoding. This was reflected by faster RTs and increased theta power at occipito-parietal and fronto-central electrodes for high compared to low reward predicting images. Importantly, both, the behavioral and oscillatory reward anticipation effect were absent in the elderly participants. However, across all elderly subjects inter-individual variance in reward related RT benefits were predicted by the integrity of the dopaminergic midbrain, suggesting a close link.

SN/VTA neurons fire in response to reward-predicting cues and release dopamine into the ventral striatum and other parts of the basal ganglia. Computational models (Niv, 2007), functional imaging (Pessiglione et al., 2007; Schott et al., 2008) and pharmacological interventions (Beierholm et al., 2013) indicate that dopamine levels drive response vigor. More precisely, phasic dopamine transmission in response to a reward-predicting cue results in faster RTs (Satoh et al., 2003; Ko and Wanat, 2016), probably via innervating the NAcc (McGinty et al., 2013), which might serve as hub between reward related limbic signals and motor control circuits (Groenewegen et al., 1996). Therefore, movement initiation relies on a healthy dopaminergic system and, consequently, slower RTs have been shown in patients with Parkinson disease, who suffer from a massive loss of dopamine neurons (Mazzoni et al., 2007), during pharmacologically induced dopamine depletion in rats (Cole and Robbins, 1989), as well as in humans after D2 receptor blockage by haloperidol (Saeedi et al., 2006; Veselinović et al., 2013).

Our data resonate well with these previous findings, demonstrating that higher reward led to accelerated responses in young participants, probably due to increased dopamine transmission from the SN/VTA. Importantly, this pattern was absent in the elderly, and, across subjects, SN/VTA integrity (MT, reflecting myelin content) predicted the degree of reward related RT benefit. Physiologically, myelin covers neural axons and plays a key role in transmitting electrical signals (Nave and Werner, 2014; Pajevic et al., 2014). During healthy aging, it tends to become more vulnerable (Bartzokis, 2004), resulting in a degeneration of myelin sheaths (Peters, 2002; Draganski et al., 2011; Callaghan et al., 2014). In the light of these observations, our findings suggest that age related myelin loss within the SN/VTA might impair reward related signal transmission. This proposed relationship of structural and functional variability is further underlined by a recent study showing that the volume of the dopaminergic midbrain (as measured with structural MRI) relates to reward induced reactivity of the NAcc (as measured with fMRI), which in turn links to the amplitude of the reward related feedback negativity (as measured with EEG; Carlson et al., 2015).

In the present study, reward related RT benefit in the young participants was paralleled by increased theta power for high vs. low reward, which is compatible with previous findings (Gruber et al., 2013). While our EEG data do not allow conclusions about the precise neuronal sources, theta oscillations in general are suggested to play a major role in several cognitive functions including goal-directed behavior and memory processes by synchronizing neuronal activity across limbic, striatal and cortical nodes, probably reflecting the coordination of processing relevant information (Buzsáki and Draguhn, 2004; Düzel et al., 2010; Herweg et al., 2016). Since theta power did not directly relate to the integrity of SN/VTA or NAcc, and since there was no relationship to RTs in our current work, it is also plausible that it might more closely reflect reward processing in cortical brain regions such as the medial frontal cortex (Silvetti et al., 2014).

Alternatively, theta oscillations may relate to changes in hippocampal activity and associated formations of new memories (Axmacher et al., 2006; Colgin, 2016). Therefore, increased theta power in the high reward condition may not solely resemble reward processing, but also (indirectly) reflect enhanced encoding activity within the hippocampus and interconnected brain regions, due to reward related dopaminergic innervations. However, in contrast to our hypothesis and contradictory to other studies (Gruber et al., 2013), reward did not improve subsequent recognition in the young or elderly participants. This might be explained by the theory that reward related dopaminergic neuromodulation affects late LTP, but not, or to a lower degree, early LTP (Lisman et al., 2011). Therefore, the time between encoding and recognition might have been too short for an effect of reward on memory consolidation.

Previous fMRI studies indicate that, in contrast to the young, elderly participants do not exhibit mesolimbic reward prediction signals but preserved activation to reward feedback (Schott et al., 2007; Samanez-Larkin et al., 2014). In contrast to these observations and our initial hypothesis (see “Introduction” Section), we did not find reward related outcome signals in the elderly. However, this pattern fits other EEG studies, reporting reduced electrophysiological activity in response to reward outcome in the elderly (Hämmerer et al., 2011).

Finally, we would like to point out the following limitations of our study: first, MT is only an indirect marker of myelin, even though it closely correspond to its biochemical counterpart (Schmierer et al., 2004). Second, further research has to determine whether the structure-behavior link differs between age groups, as structural data for the young participants were not collected in this study. Third, it may be possible that the elderly had controlled for interference by focusing their attention on the central picture, neglecting the surrounding frame (Maylor and Lavie, 1998)—further studies may control for this by using a pre-stimulus cue. Fourth, due to the use of a cross-sectional and not a longitudinal design, the term “age related difference” (or similar phrases) relates to group differences between young and elderly participants and not to individual development over time.

## Conclusion

Anticipating reward invigorates behavior in younger subjects as reflected in faster RTs and changes in theta power. These effects could not be observed in the elderly, which is compatible with the notion of impaired abilities to learn predictive value of reward cues. Importantly, across all elderly subjects, the structural integrity of the SN/VTA predicted a RT benefit by reward. As such, our data further underline a close relationship between theta oscillations and reward processing, and they suggest that declines of the dopaminergic mesolimbic system directly relates to changes in motivated behavior by reward during healthy aging.

## Author Contributions

TKS acquired the data. TKS and NB designed the study, analyzed the data and wrote the article.

## Conflict of Interest Statement

The authors declare that the research was conducted in the absence of any commercial or financial relationships that could be construed as a potential conflict of interest.
